# Diagnostic value of active protraction and retraction for sternoclavicular joint pain

**DOI:** 10.1186/1471-2474-15-421

**Published:** 2014-12-11

**Authors:** Alexander Van Tongel, Anne Karelse, Bart Berghs, Tom Van Isacker, Lieven De Wilde

**Affiliations:** Department of Orthopaedic Surgery and Traumatology, Ghent University Hospital, De Pintelaan 185, B-9000 Gent, Belgium; Department of Orthopaedics and Traumatology, Upper Limb Unit, AZ Sint-Jan AV Brugge - Oostende, Campus Brugge, Ruddershove 1, B-8000 Brugge, Belgium; Department of Orthopaedics and Traumatology, Zorgsaam Terneuzen Wielingenlaan 2, 4535 PA Terneuzen, The Netherlands; Department of Orthopaedics and Traumatology, AZ Sint-Lucas Brugge, Sint Lucaslaan 29, B 8310 Brugge, Belgium

**Keywords:** Clinical test, Sternoclavicular, Arthopathy, Sensitivity, Specificity, Scapular protraction

## Abstract

**Background:**

Sternoclavicular joint (SCJ) arthropathy is an uncommon cause of mechanical pain. The aim of this study is to evaluate the diagnostic value of two active clinical tests for localizing the sternoclavicular joint as the source of mechanical pain.

**Methods:**

All patients between June 2011 and October 2013 that visited the orthopedic departments of three hospitals with atraumatic pain in the area of the SC joint were evaluated. Local swelling, pain at palpation, pain during arm elevation and two newly described tests (pain during active scapular protraction and retraction) were evaluated. CT images were evaluated. The patients were then divided into two groups according to whether they had a ≥50% decrease in pain following the SCJ injection. Sensitivity and specificity for local swelling, the four clinical tests and CT-scan were measured.

**Results:**

Forty eight patients were included in this study and SC joint pain was confirmed in 44. The tests with highest sensitivity were pain on palpation, (93% sensitivity) and pain during active scapular protraction (86%). CT-scan showed a sensitivity of 84%. Local swelling showed a high specificity (100%).

**Conclusion:**

Pain at the SCJ during active scapular protraction is a good clinical diagnostic tool for SC arthropathy.

**Electronic supplementary material:**

The online version of this article (doi:10.1186/1471-2474-15-421) contains supplementary material, which is available to authorized users.

## Background

Joint pain (pain localized in the area of a joint) is clinically reproducible by pain provocation tests, and, ideally, is completely relieved by infiltration of the symptomatic joint with local anesthetics. Sternoclavicular joint (SCJ) pain is uncommon but the joint is subject to the same disease processes that occur in other joints.

Arthritis is the most common nontraumatic disease of the SCJ. The most frequent types of arthritic illnesses include post-traumatic, septic, inflammatory seropositive (rheumatoid arthritis), seronegative (ankylosing spondylitis, Reiter syndrome, colitic, and psoriatic), and crystal (gout, pseudogout). Other less common SCJ-specific arthritides include Friedrich disease, condensing osteitis, SAPHO (synovitis, acne, pustulosis, hyperostosis, and osteitis), and palmoplantar pustulosis. Neoplasms involving the SCJ include primary tumors, such as Ewing sarcoma, and secondary neoplasm, such as squamous cell carcinoma and adenocarcinoma [1].

Patients are mostly seen for consultation because of localized swelling and/or mechanical pain at the level of the SCJ [2–4]. However, pathology of the SCJ can also cause referred pain to areas distant from the joint. These area includes the anterior trapezial fold, along the lateral clavicle to the anterior shoulder, the neck and jaw and it can overlap the pain pattern of those of the acromioclavicular (AC) joint, the subacromial space and cervical nerves [5–7].

The most common used imaging technique to evaluate SCJ pathology is computertomography (CT) [8–10]. The sensitivity of local swelling and pain during palpation at the SCJ has been evaluated [1]. But in contrast to commonly described active tests of the shoulder girdle [11–14], to our knowledge, no active clinical tests of the joint has not been evaluated. The aim of this study is to evaluate the diagnostic value of two newly described active clinical test for localizing the SCJ as cause of the mechanical pain.

## Methods

All patients that visited the orthopedics departments of three hospitals between June 2011 and June 2013 with a history of atraumatic pain in the area of the SC joint were included in this retrospective study.

**Figure 1:**
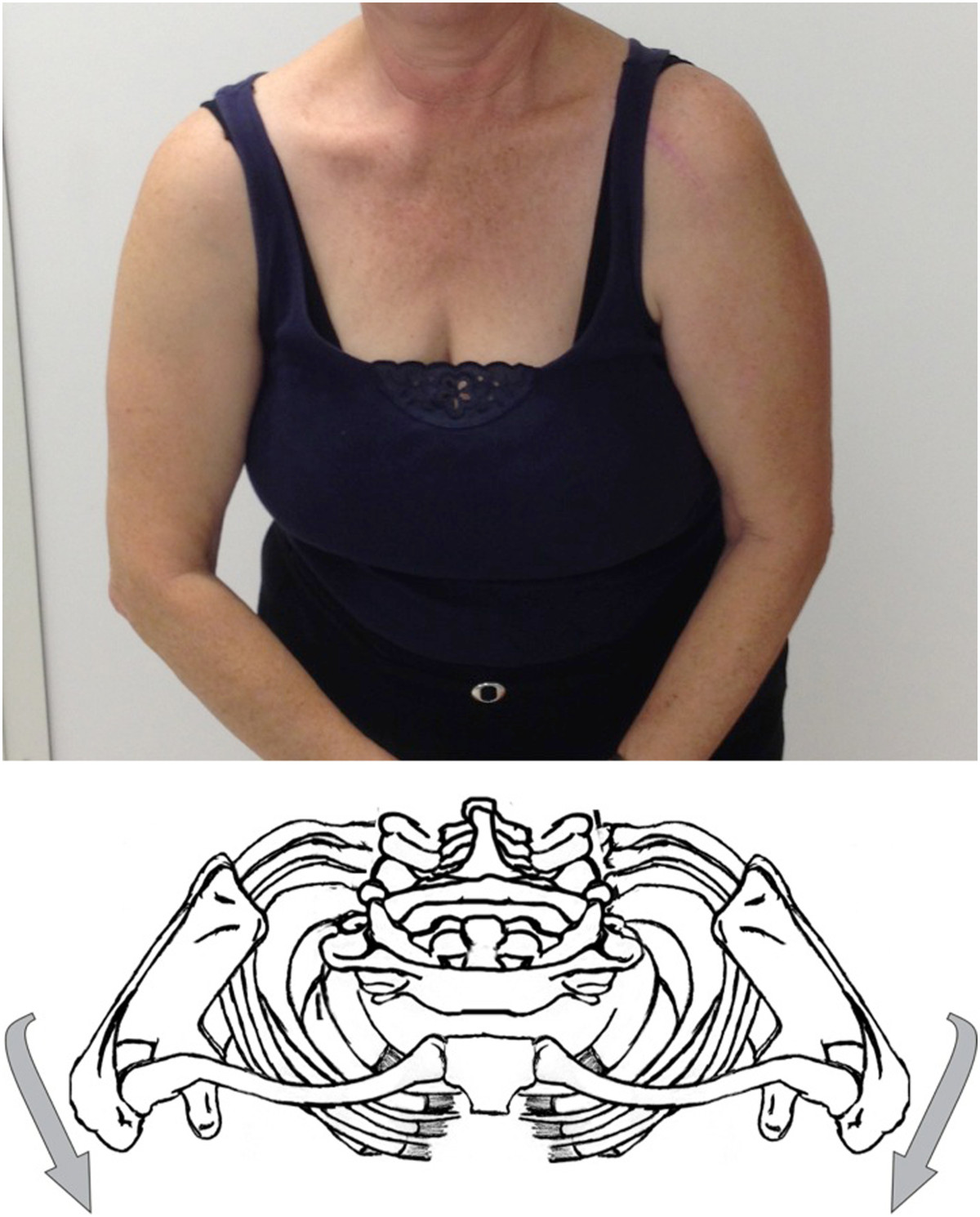
**Active scapular protraction.**

**Figure 2:**
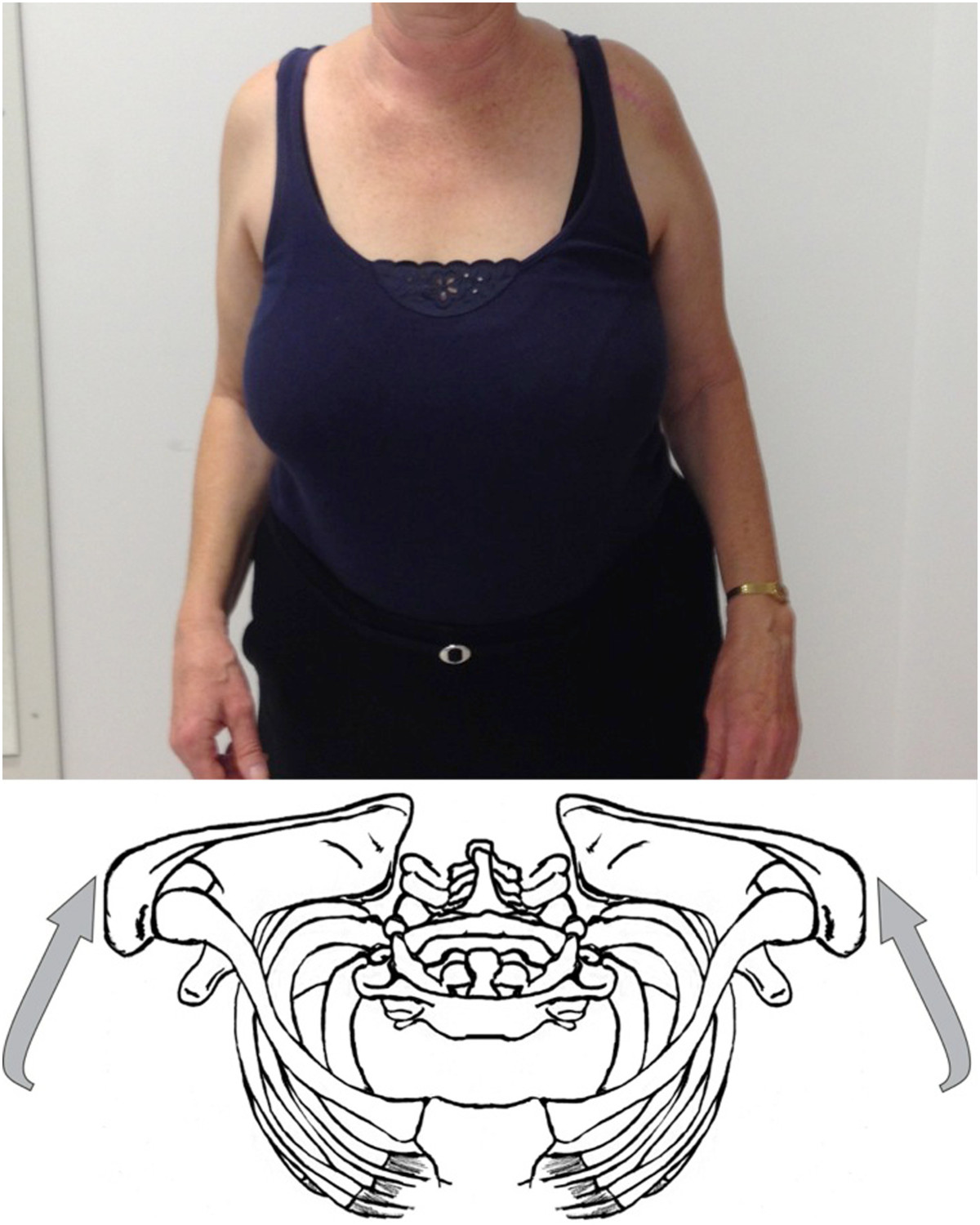
**Active scapular retraction.**

Exclusion criteria were as follows: previous SC surgery, (2) atraumatic SC instability, (3) previous or known allergies to lidocaine and (4) refusal of a reference standard injection lidocaine test. Age, sex, hand dominance, affected arm, onset and duration of pain are documented.All patients were examined bilaterally in upright and supine position using a standard protocol. This included inspection and clinical examination of the neck and shoulder region. Also signs of palmar and plantar pustulosis, acne or infection typical of palmoplantar pustulosis, SAPHO syndrome, and septic arthritis were evaluated. Local swelling of the SCJ and pain on palpation of the SCJ and ACJ. Active and passive range of motion of the neck were measured. With regard to the shoulder, active and passive forward flexion, abduction, internal and external rotations were measured by visual estimation. If active flexion above 100° was painful at the SC joint area, the test was positive. Next the patient was asked to actively perform maximal protraction and retraction of the shoulder girdle in an upright position and pain during these tests was evaluated (Figures 1 and 2).

### Imaging studies

All patients were positioned in the CT gantry according to a previously described method, that is in dorsal recumbency, with a cushion on the belly and a strap around the body and this cushion, to keep the arm adducted in the coronal plane and the forearm flexed in the sagittal plane of the body [15]. This standardized position mimics a reproducible surgical position and minimizes positional errors. All sternoclavicular joints were examined with CT scans with following settings: Somatom Volume Zoom – Siemens (Siemens Business Park, Marie Curiesquare 30 - Square Marie Curie 30; 1070 Brussel – Bruxelles) ; matrix: 512/ kV:140/ eff. mAs: 350. The scan field of view (SFOV) was always 500. Radiographic parameters of rheumatoid or osteoarthritis (bone cyst, osteophytes, narrowing of the joint space, anterior subluxation), hyperostosis of the sternum, clavicles, and upper ribs (sternocostoclavicular hyperostosis (SCCH)), obliteration of the marrow space (condensing osteitis), irregularity of SCJ with bony destruction of medial end of clavicle (friederich disease) or joint effusion, bone and cartilage erosions, gas within the joint, and soft tissue swelling (septic arthritis) or no abnormality were checked on the CT.

### Reference test standard

The reference test standard was an ultrasound guided SCJ infiltration, of 1 mL of 2% (v/v) lidocaine [16]. All patients with pain in the sternoclavicular area were injected by an experienced orthopaedic surgeon (i.e., one of the authors). Patients sensing alleviation of pain with 50% or more, within ten minutes after the lidocaine injection, were considered to have SCJ pain; i.e., they have a positive reference test standard. Patients with less than 50% pain relief were considered to not have SCJ pain [17]. If there was a positive reference test is an ultrasound guided SCJ infiltration of 1 mL (40 mg) of methylprednisolone [16, 18] was given.

If no abnormality was found on CT but the patient had a positive reference standard test, the the pathology was called monoarthritis e causa ignota.

### Statistical analysis

Sensitivity, specificity, positive and negative predictive values of the clinical diagnostic tests were determined with the methods described by Sackett et al. [19].

This study was approved by the appropriate ethics committee (UZ Ghent, Chairman: Prof. Dr.Rubens Registration number: B670201317780).

## Results

### Patient demographics

Between June 2011 and June 2013, 48 patients with unilateral atraumatic pain in the area of the SC who met our inclusion criteria were enrolled in this study (Table 1).

**Table 1:** **Sensitivity, specificity and predictive value of clinical and imaging test**

	Sensitivity	Specificity	Positive predictive value	Negative predictive value
Tenderness	93%	25%	93%	25%
Prominence	61%	100%	100%	0%
Pain active protraction (N)	86%	50%	95%	25%
Pain active retraction (N)	45%	75%	95%	11%
Pain active elevation	84%	50%	93%	14%
CT	84%	100%	100%	0%

There were 9 men and 39 women, with an average age of 60 years, ranging from 23 to 74 years. Forty-one patients were right-handed and the dominant shoulder was involved in 40, while the nondominant arm was affected in 8. All patients reported an insidious onset of symptoms. Average duration of symptoms was 11 months (range, 3–24).

### Sternoclavicular arthropathy

Forty-four patients were confirmed to have SCJ abnormality by their response to the SCJ injection. Thus, the prevalence of SCJ abnormality in this patient population was 90%. This high prevalence indicates that mechanical pain in the area of the sternoclavicular region is itself a reliable sign for SCJ arthropathy. Four patients had no relief of pain after the injection.

These positive and negative (control) groups were compared with regard to their responses to the clinical and imaging tests.

**Figure 3:**
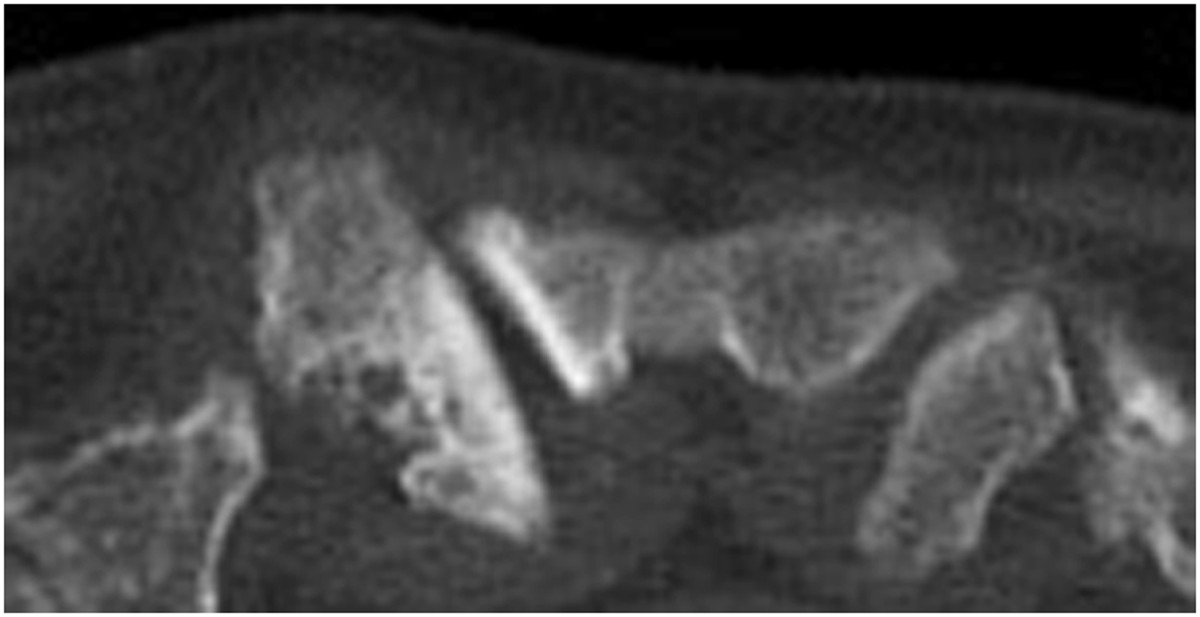
**Axial CT image in patients with symptomatic degenerative SC arthritis.**

The most sensitive clinical test for identifying SCJ arthropathy was examination for local SCJ tenderness (93% sensitivity), followed by the pain during active protraction (86%). CT showed a sensitivity of 84% (Table 1). Four different pathologies were diagnosed (Table 2) (Figure 3).

**Table 2:** **Different diagnosis of sternoclavicular arthropathy**

Diagnosis	Number of patients
Degenerative arthritis	33
SCCH	2
Freiderich disease	2
Monoarthtritis e cause ignota	7

## Discussion

SC arthropathy is an uncommon cause of pain. The location of pain originating from the SC joint can be diverse and patients are often not able to identify the exact location. As stated by Hassett et al., the pain pattern can overlap those of the AC joint, the subacromial space and cervical nerves. Hence, SC joint pain needs to be considered in the differential diagnosis of pain from these structures and their referred anatomical site [6]. But because of its rarity, accurate diagnosis is often delayed. Easy clinical tests of the joint that can be included in the normal shoulder exam could help to obtain a sooner diagnosis and treatment for patients with SC arthropathy.

Three clinical signs for SC arthropathy have previously been described in the literature: tenderness by palpation, a local swelling and pain during active elevation [20–23]. In our study, local tenderness was the most sensitive test for SC arthropathy (93%). Also, in patients with transient SC arthropathy, tenderness was also a common finding (84%) [1]. In our population, local swelling was most commonly seen in patients with signs of degenerative arthritis on CT. This correlated with the findings of Van Tongel et al. who described an anterior subluxation of the clavicle in patients with symptomatic degenerative arthritis [24]. This hard prominence is in contrast with the temporary local swelling in patients with transient SC arthropathy without any signs of degeneration [1].

The high specificity of clavicular prominence for SC pathology in our population is probably not correct because, for example, bone tumors also can be a cause of prominence of the medial clavicle; however, we did not see those patients in our clinic.

Pain in the SC region during arm elevation above 100° showed a high sensitivity (84%). But a problem with this test is that it requires the combined motion of the SC, AC, glenohumeral, and scapulothoracic joints. This means that in some cases of glenohumeral pathology (frozen shoulder, pseudoparalysis) this test cannot be used. This is a reason why we described the active scapular protraction and retraction. In our opinion, this motion tries to isolate the SC motion as much as possible. Inman et al. described that during scapular protraction and retraction of the shoulders, no appreciable motion occurs at the AC joint or any great rotation of the clavicle. He stated that scapular protraction and retraction is occurring predominantly at the SCJ [25]. Abott et al. described that during retraction and protraction of the shoulder the scapula describes an arc of 50 degrees around the SCJ. In the average individual the clavicular motion is 35 degrees at the SCJ and scapular movement at the AC joint accounts for 15 degrees [26]. By performing active scapular protraction and retraction the motion at the glenohumeral joint and the AC joint is minimized.

Anatomical study of the SCJ showed that the articular surface of the clavicle is located antero-inferiorly [27]. This means active scapular protraction creates a compression and active scapular retraction a distraction of the joint.

Scapular protraction showed a high sensitivity (91%) in contrast to scapular retraction (39%). Similar to the clinical test for the AC joint it seems that compression of the SC joint is more sensitive to diagnose SC arthropathy than distraction [28].

The most commonly used technical investigation to evaluate the SCJ is CT-scan. CT imaging has been described to be superior to other imaging techniques when evaluating narrowing of the joint space, osteophytes, subchondral sclerosis, and cysts at both sides of the joint [3–5, 21, 29]. Conventional plain x-rays of the joint may be suboptimal to evaluate abnormalities because the overlying ribs, spinal column, and soft-tissue obscure joint detail [30]. MRI should be the first modality of choice when suspecting malignancies or infection in the SC region, but not for the diagnosis of degenerative osteoarthritis [31]. Scintigraphy is a sensitive method for detecting disorders with increased bone turnover, but is in general not specific for disorders in the SC region except for the “bullhead-like” sign in sternocostoclavicular hyperostosis [31].

In 86% of our cases, CT showed pathological changes. In 14% no pathology was seen on CT scan. Two weeks after the infiltration, these patients were asymptomatic and no further technical investigation was performed. These patients were diagnosed with a mono-arthritis e causa ignota.

This study is unique because, to our knowledge, no one has previously described active isolated thoracoscapular to evaluate SC arthropathy. This is also the first time the values of different tests used to diagnose SCJ pain is evaluated. Our study also has several weaknesses. First, the study group consisted of a relatively small number of patients who fulfilled the inclusion criteria. Second, several other causes of SC arthropathy (seronegative spondyloarthropathie, septic arthritis, rheumatoid artritis) were not seen in our patient population.

## Conclusion

Pain during active scapular protraction is an easy clinical test that can be a good clinical diagnostic tool for sternoclavicular arthopathy

## Consent

Written informed consent was obtained from the patient for the publication of this report and any accompanying images.

## Authors’ original submitted files for images

Below are the links to the authors’ original submitted files for images. Authors’ original file for figure 1 Authors’ original file for figure 2 Authors’ original file for figure 3

## Abbreviations

SC: Sternoclavicular
SCJ: Sternoclavicular joint
CT: Computertomography
AC: Acromioclavicular.
