# Supplementary figures and images for: Diagnostic value of active protraction and retraction for sternoclavicular joint pain
Source: BMC Musculoskelet Disord. 2014 Dec 11;15:421. doi: 10.1186/1471-2474-15-421 (PMC4295279; doi:10.1186/1471-2474-15-421)

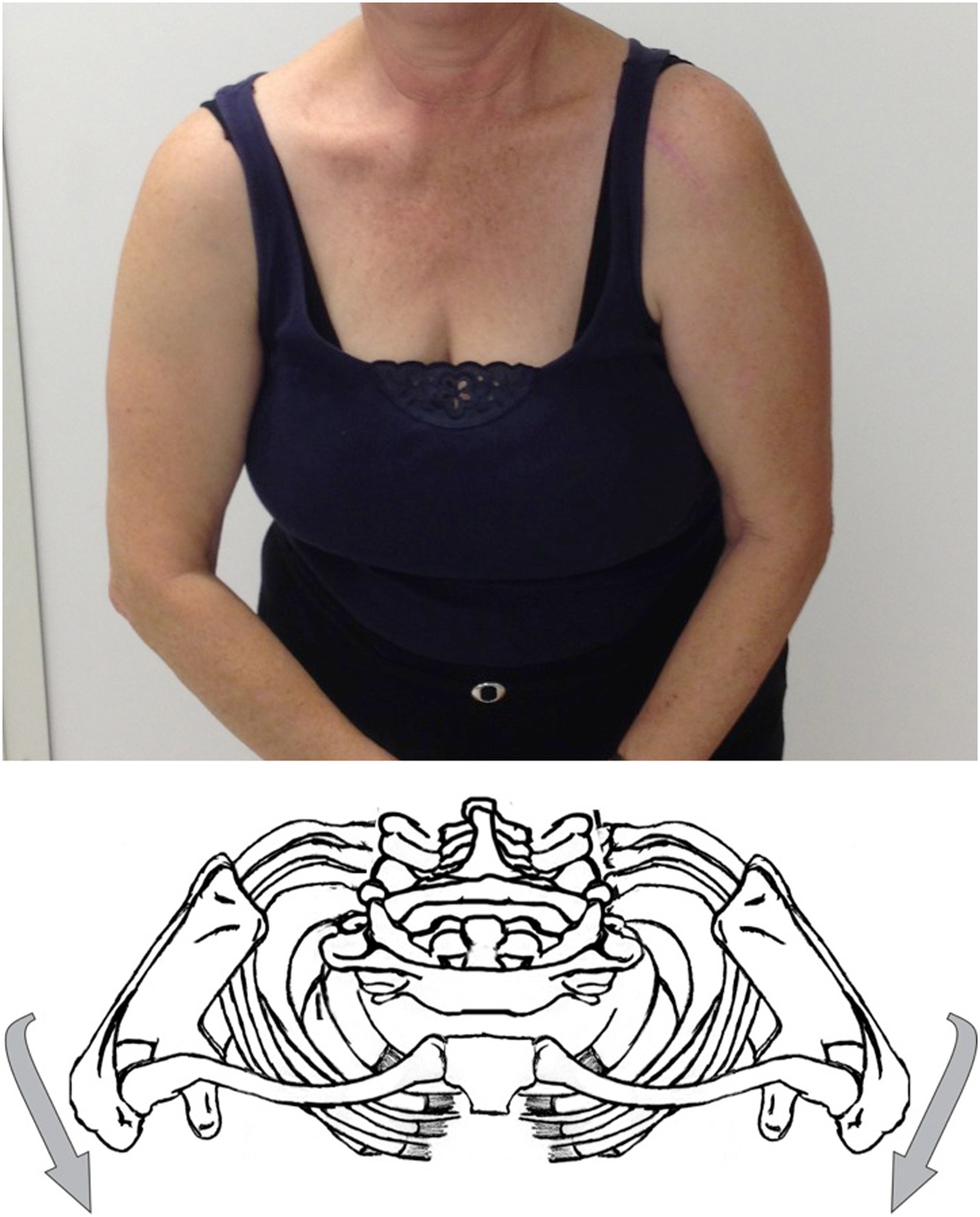

Supplement: Supplementary file 1 — Authors’ original file for figure 1 [file 12891_2014_2347_MOESM1_ESM.tif]

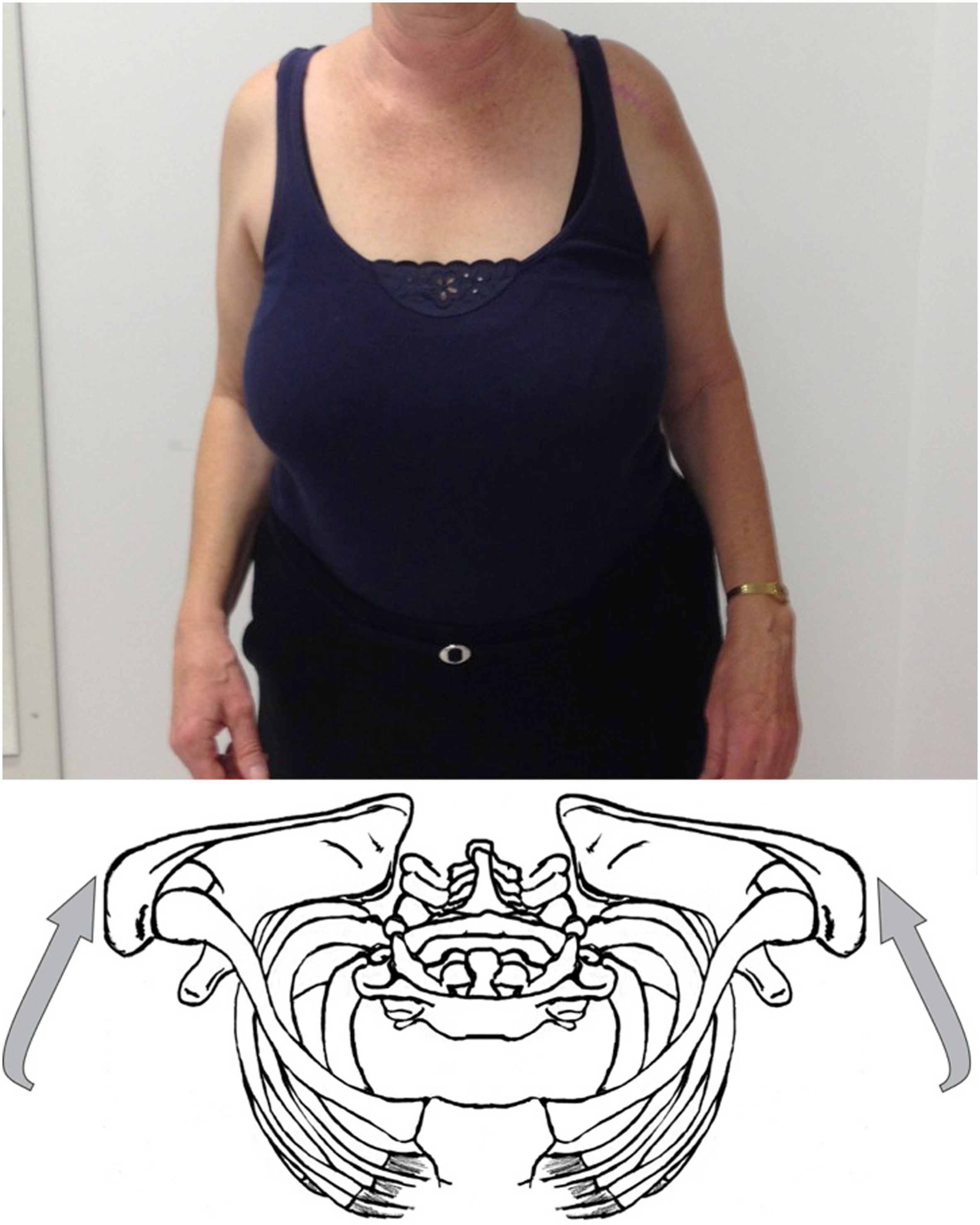

Supplement: Supplementary file 2 — Authors’ original file for figure 2 [file 12891_2014_2347_MOESM2_ESM.tif]

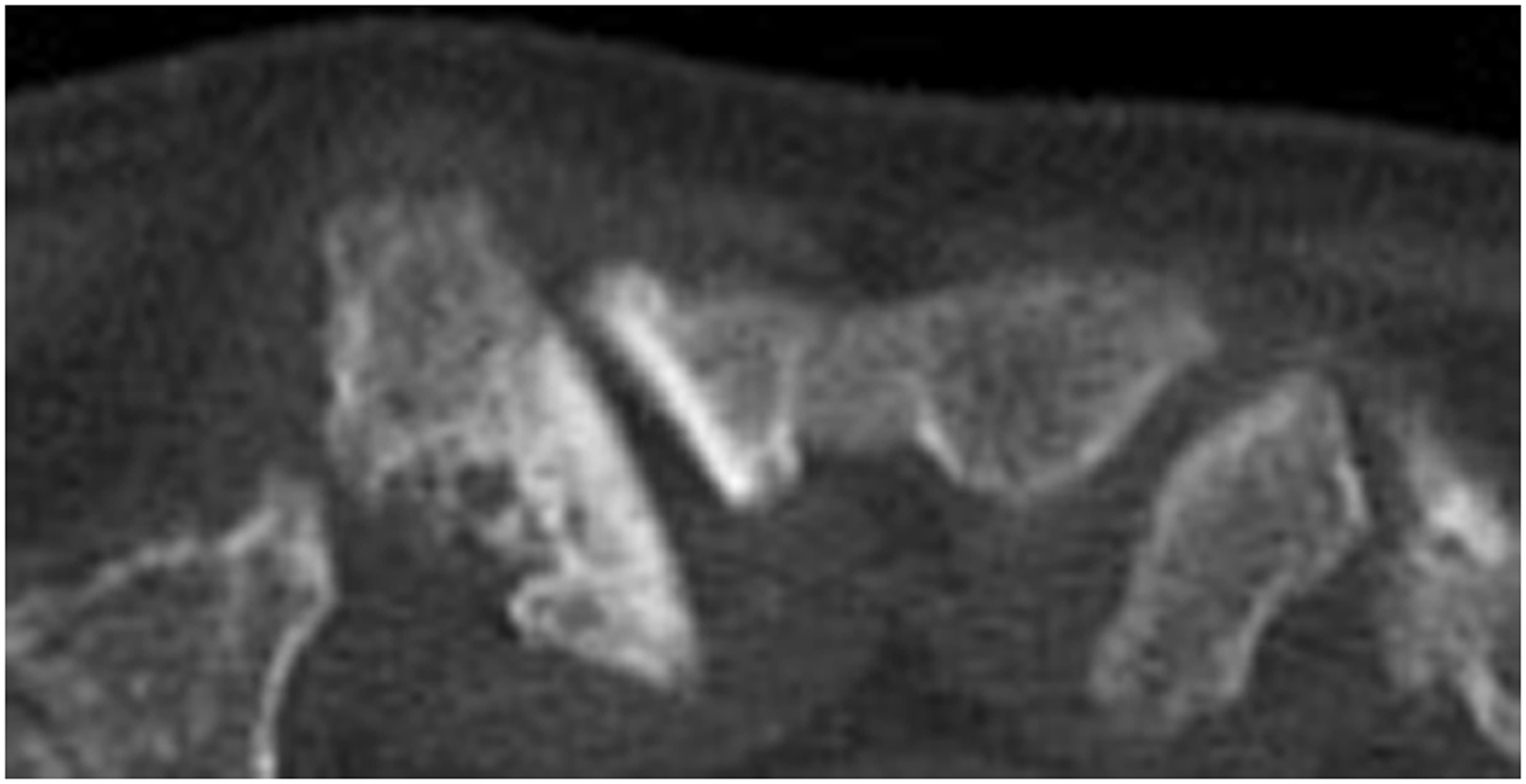

Supplement: Supplementary file 3 — Authors’ original file for figure 3 [file 12891_2014_2347_MOESM3_ESM.tif]
